# 5-(Pyridinium-4-yl)-1*H*-1,2,3,4-tetra­zol-1-ide

**DOI:** 10.1107/S1600536810050257

**Published:** 2010-12-08

**Authors:** Qian Xu, Jie Xu

**Affiliations:** a Ordered Matter Science Research Center, College of Chemistry and Chemical Engineering, Southeast University, Nanjing 210096, People’s Republic of China

## Abstract

In the title zwitterionic mol­ecule, C_6_H_5_N_5_, the tetra­zole and pyridine rings are nearly coplanar, making a dihedral angle of 2.08 (1)°. In the crystal, mol­ecules are connected by classical N—H⋯N and weak C—H⋯N hydrogen bonds.

## Related literature

For applications of tetra­zole derivatives, see: Zhao *et al.* (2008 ▶); Fu *et al.* (2008 ▶, 2009 ▶). For the crystal structures and properties of related compounds, see: Fu *et al.* (2007 ▶, 2009 ▶); Fu & Xiong (2008 ▶).
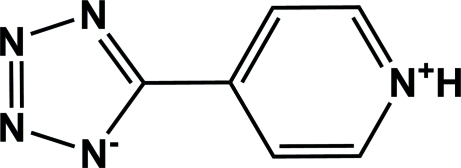


## Experimental

### 

#### Crystal data

C_6_H_5_N_5_
*M* *_r_* = 147.15
Monoclinic, 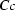

*a* = 7.0508 (14) Å
*b* = 7.4007 (15) Å
*c* = 11.926 (2) Å
β = 96.56 (3)°
*V* = 618.2 (2) Å^3^
*Z* = 4
Mo *K*α radiation
μ = 0.11 mm^−1^
*T* = 298 K
0.30 × 0.20 × 0.15 mm

#### Data collection

Rigaku Mercury2 diffractometer
3122 measured reflections
719 independent reflections
633 reflections with *I* > 2σ(*I*)
*R*_int_ = 0.039

#### Refinement

*R*[*F* ^2^ > 2σ(*F* ^2^)] = 0.040
*wR*(*F* ^2^) = 0.096
*S* = 1.13
719 reflections
100 parameters
2 restraints
H-atom parameters constrained
Δρ_max_ = 0.23 e Å^−3^
Δρ_min_ = −0.21 e Å^−3^

### 

Data collection: *CrystalClear* (Rigaku, 2005 ▶); cell refinement: *CrystalClear*; data reduction: *CrystalClear*; program(s) used to solve structure: *SHELXTL* (Sheldrick, 2008 ▶); program(s) used to refine structure: *SHELXTL*; molecular graphics: *SHELXTL*; software used to prepare material for publication: *SHELXTL*.

## Supplementary Material

Crystal structure: contains datablocks I, global. DOI: 10.1107/S1600536810050257/xu5093sup1.cif

Structure factors: contains datablocks I. DOI: 10.1107/S1600536810050257/xu5093Isup2.hkl

Additional supplementary materials:  crystallographic information; 3D view; checkCIF report

## Figures and Tables

**Table 1:** Hydrogen-bond geometry (Å, °)

*D*—H⋯*A*	*D*—H	H⋯*A*	*D*⋯*A*	*D*—H⋯*A*
N1—H1*A*⋯N2^i^	0.86	1.89	2.745 (4)	176
N1—H1*A*⋯N3^i^	0.86	2.52	3.306 (4)	152
C1—H1⋯N5^ii^	0.93	2.46	3.308 (4)	152
C5—H5⋯N4^iii^	0.93	2.38	3.168 (4)	142

Symmetry codes: (i) 
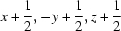
; (ii) 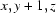; (iii) 
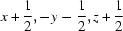
.

**Fig. 1.**
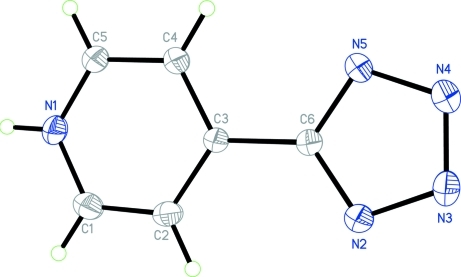
A view of the title compound with the atomic numbering scheme. Displacement ellipsoids were drawn at the 30% probability level.

## supplementary crystallographic information

### Comment

Tetrazole compounds attracted more attention as phase transition dielectric materials for its application in micro-electronics, memory storage. With the purpose of obtaining phase transition crystals of tetrazole compound, a series of new materials have been elaborated with this organic molecule (Zhao *et al.*, 2008; Fu *et al.*, 2008; Fu *et al.*, 2007; Fu & Xiong 2008). We report here the crystal structure of the title compound, 5-(pyridinium-4-yl)tetrazol-1-ide.

The dielectric constant of title compound as a function of temperature indicates that the permittivity is basically temperature-independent, suggesting that this compound should be not a real ferroelectrics or there may be no distinct phase transition occurred within the measured temperature range. Similarly, below the melting point (413K) of the compound, the dielectric constant as a function of temperature also goes smoothly, and there is no dielectric anomaly observed (dielectric constant equaling to 6.1 to 7.9).

In the title compound (Fig.1), the pyridine N atom is protonated, thus indicating a positive charge in the pyridine N atom. And the tetrazole ring was showing a negative charge to make the charge balance. The tetrazole and pyridine rings are twisted from each other by a dihedral angle of 2.08 (1)°. The geometric parameters of the tetrazole rings are comparable to those in related molecules (Fu *et al.*, 2009).

In the crystal structure the molecules are connected by classic N—H···N and weak C—H···N hydrogen bonds (Table 1).

### Experimental

5-(Pyridinium-4-yl)tetrazol-1-ide was obtained commercially, and the single crystals were obtained from an ethanol solution.

### Refinement

H atoms attached to N atoms were located in a difference Fourier map, and refined in riding mode with N–H = 0.86 Å and U*_iso_*(H) = 1.2U*_eq_*(N). Other H atoms were fixed geometrically and treated as riding with C–H = 0.93 Å and U*_iso_*(H) = 1.2U*_eq_*(C). As no significant anomalous scattering, Friedel pairs were merged.

### Crystal data

**Table:**

C_6_H_5_N_5_	*F*(000) = 304
*M**_r_* = 147.15	*D*_x_ = 1.581 Mg m^−^^3^
Monoclinic, *C**c*	Mo *K*α radiation, λ = 0.71073 Å
Hall symbol: C -2yc	Cell parameters from 1425 reflections
*a* = 7.0508 (14) Å	θ = 3.4–24.5°
*b* = 7.4007 (15) Å	µ = 0.11 mm^−^^1^
*c* = 11.926 (2) Å	*T* = 298 K
β = 96.56 (3)°	Block, colorless
*V* = 618.2 (2) Å^3^	0.30 × 0.20 × 0.15 mm
*Z* = 4	

### Data collection

**Table:**

Rigaku Mercury2 diffractometer	633 reflections with *I* > 2σ(*I*)
Radiation source: fine-focus sealed tube	*R*_int_ = 0.039
graphite	θ_max_ = 27.5°, θ_min_ = 3.4°
Detector resolution: 13.6612 pixels mm^-1^	*h* = −9→9
CCD profile fitting scans	*k* = −9→9
3122 measured reflections	*l* = −15→15
719 independent reflections	

### Refinement

**Table:**

Refinement on *F*^2^	Primary atom site location: structure-invariant direct methods
Least-squares matrix: full	Secondary atom site location: difference Fourier map
*R*[*F*^2^ > 2σ(*F*^2^)] = 0.040	Hydrogen site location: inferred from neighbouring sites
*wR*(*F*^2^) = 0.096	H-atom parameters constrained
*S* = 1.13	*w* = 1/[σ^2^(*F*_o_^2^) + (0.056*P*)^2^] where *P* = (*F*_o_^2^ + 2*F*_c_^2^)/3
719 reflections	(Δ/σ)_max_ < 0.001
100 parameters	Δρ_max_ = 0.23 e Å^−^^3^
2 restraints	Δρ_min_ = −0.21 e Å^−^^3^

### Special details

**Table:**

Geometry. All esds (except the esd in the dihedral angle between two l.s. planes) are estimated using the full covariance matrix. The cell esds are taken into account individually in the estimation of esds in distances, angles and torsion angles; correlations between esds in cell parameters are only used when they are defined by crystal symmetry. An approximate (isotropic) treatment of cell esds is used for estimating esds involving l.s. planes.
Refinement. Refinement of F^2^ against ALL reflections. The weighted R-factor wR and goodness of fit S are based on F^2^, conventional R-factors R are based on F, with F set to zero for negative F^2^. The threshold expression of F^2^ > 2sigma(F^2^) is used only for calculating R-factors(gt) etc. and is not relevant to the choice of reflections for refinement. R-factors based on F^2^ are statistically about twice as large as those based on F, and R- factors based on ALL data will be even larger.

### Fractional atomic coordinates and isotropic or equivalent isotropic displacement parameters (Å^2^)

**Table:**

	*x*	*y*	*z*	*U*_iso_*/*U*_eq_	
N1	1.0961 (4)	0.3035 (4)	0.4442 (2)	0.0382 (6)	
H1A	1.1462	0.3791	0.4937	0.046*	
N2	0.7525 (4)	−0.0341 (4)	0.1099 (2)	0.0375 (6)	
N3	0.7094 (4)	−0.1968 (3)	0.0653 (2)	0.0443 (7)	
N4	0.7884 (4)	−0.3224 (3)	0.1321 (2)	0.0444 (8)	
N5	0.8864 (3)	−0.2457 (4)	0.2218 (2)	0.0395 (7)	
C1	0.9963 (5)	0.3659 (4)	0.3504 (3)	0.0397 (9)	
H1	0.9805	0.4896	0.3394	0.048*	
C2	0.9185 (4)	0.2487 (4)	0.2718 (3)	0.0368 (7)	
H2	0.8496	0.2920	0.2062	0.044*	
C3	0.9406 (4)	0.0634 (4)	0.2879 (2)	0.0290 (6)	
C4	1.0442 (4)	0.0050 (4)	0.3861 (2)	0.0372 (8)	
H4	1.0622	−0.1179	0.3996	0.045*	
C5	1.1202 (4)	0.1280 (4)	0.4632 (3)	0.0391 (7)	
H5	1.1896	0.0888	0.5298	0.047*	
C6	0.8605 (4)	−0.0704 (4)	0.2067 (2)	0.0305 (7)	

### Atomic displacement parameters (Å^2^)

**Table:**

	*U*^11^	*U*^22^	*U*^33^	*U*^12^	*U*^13^	*U*^23^
N1	0.0486 (14)	0.0342 (16)	0.0299 (14)	−0.0035 (13)	−0.0037 (10)	−0.0059 (13)
N2	0.0465 (15)	0.0288 (12)	0.0353 (13)	0.0003 (11)	−0.0040 (10)	0.0010 (11)
N3	0.0595 (17)	0.0348 (13)	0.0359 (13)	−0.0060 (15)	−0.0060 (11)	−0.0082 (14)
N4	0.062 (2)	0.0293 (14)	0.0404 (19)	−0.0004 (13)	−0.0010 (15)	−0.0045 (12)
N5	0.0533 (16)	0.0251 (13)	0.0377 (16)	0.0014 (11)	−0.0051 (13)	−0.0026 (11)
C1	0.0455 (17)	0.029 (2)	0.0431 (16)	0.0041 (15)	0.0005 (13)	0.0020 (15)
C2	0.0422 (17)	0.0311 (16)	0.0346 (16)	0.0031 (13)	−0.0059 (12)	0.0052 (13)
C3	0.0355 (14)	0.0240 (15)	0.0264 (13)	0.0001 (12)	−0.0007 (11)	−0.0009 (11)
C4	0.0514 (19)	0.0257 (18)	0.0319 (15)	0.0008 (12)	−0.0060 (12)	−0.0004 (13)
C5	0.0512 (18)	0.0331 (16)	0.0304 (14)	0.0035 (14)	−0.0069 (13)	−0.0014 (13)
C6	0.0369 (16)	0.0263 (15)	0.0272 (14)	0.0002 (12)	−0.0016 (11)	0.0014 (13)

### Geometric parameters (Å, °)

**Table:**

N1—C5	1.325 (4)	C1—H1	0.9300
N1—C1	1.334 (5)	C2—C3	1.391 (4)
N1—H1A	0.8600	C2—H2	0.9300
N2—C6	1.335 (4)	C3—C4	1.377 (4)
N2—N3	1.337 (4)	C3—C6	1.453 (4)
N3—N4	1.306 (4)	C4—C5	1.359 (4)
N4—N5	1.333 (4)	C4—H4	0.9300
N5—C6	1.320 (4)	C5—H5	0.9300
C1—C2	1.348 (5)		
			
C5—N1—C1	121.8 (3)	C4—C3—C2	117.8 (3)
C5—N1—H1A	119.1	C4—C3—C6	118.8 (2)
C1—N1—H1A	119.1	C2—C3—C6	123.4 (2)
C6—N2—N3	104.1 (3)	C5—C4—C3	119.6 (3)
N4—N3—N2	109.6 (3)	C5—C4—H4	120.2
N3—N4—N5	109.4 (2)	C3—C4—H4	120.2
C6—N5—N4	104.9 (2)	N1—C5—C4	120.5 (3)
N1—C1—C2	119.6 (3)	N1—C5—H5	119.7
N1—C1—H1	120.2	C4—C5—H5	119.7
C2—C1—H1	120.2	N5—C6—N2	111.8 (3)
C1—C2—C3	120.5 (3)	N5—C6—C3	122.8 (2)
C1—C2—H2	119.7	N2—C6—C3	125.4 (3)
C3—C2—H2	119.7		

### Hydrogen-bond geometry (Å, °)

**Table:**

*D*—H···*A*	*D*—H	H···*A*	*D*···*A*	*D*—H···*A*
N1—H1A···N2^i^	0.86	1.89	2.745 (4)	176
N1—H1A···N3^i^	0.86	2.52	3.306 (4)	152
C1—H1···N5^ii^	0.93	2.46	3.308 (4)	152
C5—H5···N4^iii^	0.93	2.38	3.168 (4)	142

Symmetry codes: (i) *x*+1/2, −*y*+1/2, *z*+1/2; (ii) *x*, *y*+1, *z*; (iii) *x*+1/2, −*y*−1/2, *z*+1/2.
